# Establishing *in planta* haploid inducer line by edited *SiMTL* in foxtail millet (*Setaria italica*)

**DOI:** 10.1111/pbi.13584

**Published:** 2021-04-01

**Authors:** Zixiang Cheng, Yao Sun, Suhua Yang, Hui Zhi, Tao Yin, Xiaojiao Ma, Haoshan Zhang, Xianmin Diao, Yan Guo, Xinhai Li, Chuanyin Wu, Yi Sui

**Affiliations:** ^1^ Institute of Crop Sciences Chinese Academy of Agricultural Sciences Beijing China; ^2^ State Key Laboratory of Plant Physiology and Biochemistry College of Biological Sciences China Agricultural University Beijing China; ^3^ Key Laboratory of Plant Molecular Physiology Institute of Botany Chinese Academy of Sciences Beijing China

**Keywords:** Foxtail millet, CRISPR‐Cas9, haploid embryo, *SiMTL*

Foxtail millet (*Setaria italica*), a member of the Poaceae grass family, is a C4 plant that is grown in arid and semi‐arid regions as a food and fodder crop in dozens of countries including India and China. It was domesticated from the wild species green foxtail (*S. viridis*) over 8000 years ago and is closely related to several major food, feed and bioenergy grasses including maize, sorghum, sugarcane and switchgrass. Foxtail millet is small in size and its small diploid genome has been sequenced and annotated recently (Bennetzen *et al.,* 2012; Jia *et al.,* 2013), thus increasingly becoming a model for C4 plants. The doubled haploid (DH) technology accelerates stacking and screening of recombinant haplotypes in fixed genetic backgrounds (Jacquier *et al.,* 2020). Establishment of a DH production platform in foxtail millet not only makes breeding programmes more efficient but also extends the toolkit to study complex traits related to C4 crops. To the best of our knowledge, however, there has been no haploid induction system demonstrated in this crop. Here, we report induction of haploid embryo through seed, by CRISPR‐Cas9 mediated mutation of the *SiMTL* gene, which is orthologous to the maize *MATRILINEAL*/*NOT‐LIKE‐DAD*/*PHOSPHOLIPASE A* (*MTL*/*NLD*/*ZmPLA*) gene (Gilles *et al.,* 2017; Jacquier *et al.,* 2020; Kelliher *et al.,* 2017; Liu *et al.,* 2017).

Maize seed industry has been utilizing inducer lines, derived from Stock 6, to induce haploid embryo formation for decades. The mutated gene behind the major locus responsible for haploid induction (*ggi1* – gynogenesis inducer 1 – /*qhir1*‐*qtl* for haploid induction rate 1) is *MTL*/*NLD*/*ZmPLA* that encodes a pollen‐specific phospholipase (Gilles *et al.,* 2017; Kelliher *et al.,* 2017; Liu *et al.,* 2017). *MTL*/*NLD*/*ZmPLA* is conserved in cereal plants and mutation in the unique rice orthologous gene, and in the three wheat orthologous genes enabled haploid embryo induction (Liu *et al.,* 2020; Yao *et al.,* 2018). Those findings prompted us to test the foxtail millet homolog *SiMTL* (*Seita.9G376800*) that is even closer to *MTL*/*NLD*/*ZmPLA* (82.0% sequence similarity; see Figure S1 in Yao *et al.,* 2018). We employed the CRISPR‐Cas9 editing system and designed two guide RNA sequences targeting the second and fifth exon, respectively (Figure 1a). The guide RNA expression was driven by the OsU3 promoter and *Cas9* was under control of the maize *Ubiquitin‐1* promoter. We transformed the variety Ci846 using the *Agrobacterium*‐mediated method and produced 65 T_0_ plants. Sequence analysis identified 26 events with the target sites edited at least on one allele (9 for Target 1 and 17 for Target 2), of which 19 were homozygous (same edit type on two alleles) or biallelic (different edit types on two alleles) mutants. We grew T_1_ plants from five T_0_ events carrying either homozygous or biallelic mutation of *SiMTL* that results in either frameshift or early stop (Figure 1b) and observed their seed set rate. Those *simtl* plants had an average seed set rate of 33.38%, ranking from 25.30% to 40.57% (Figure 1c). The *simtl* pollens were normal in starch accumulation (Figure 1d), similar to what was seen in rice and wheat (Liu *et al.,* 2020; Yao *et al.,* 2018). We then grew T_2_ progeny from selfing of three events (#8, #14 and #15), along with wild type plants. Of 321 T_2_ plants analysed by flow cytometry, we identified nine haploids, at an averaged haploid induction rate (HIR) of 2.8%, whereas no haploid was found among 205 wild type plants (Figure 1e and f). To test ability of the inducer lines to induce haploid embryo in different female parents, a male sterile line 682A was pollinated with pollens from the line #14. Flow cytometry analysis identified six haploid plants among 230 plants from crossing, but there was no haploid plant found among 240 F_1_ plants from the cross using Ci846 wild type plants as the pollen donor (Figure 1f). Typically, the haploid plants had short stature with smaller size in all organs including leaf, panicle and anther relative to the wild type, and did not set any seed (Figure 1g). The haploid plant from crossing was morphologically similar to the female parent (Figure 1g).

**Figure 1:**
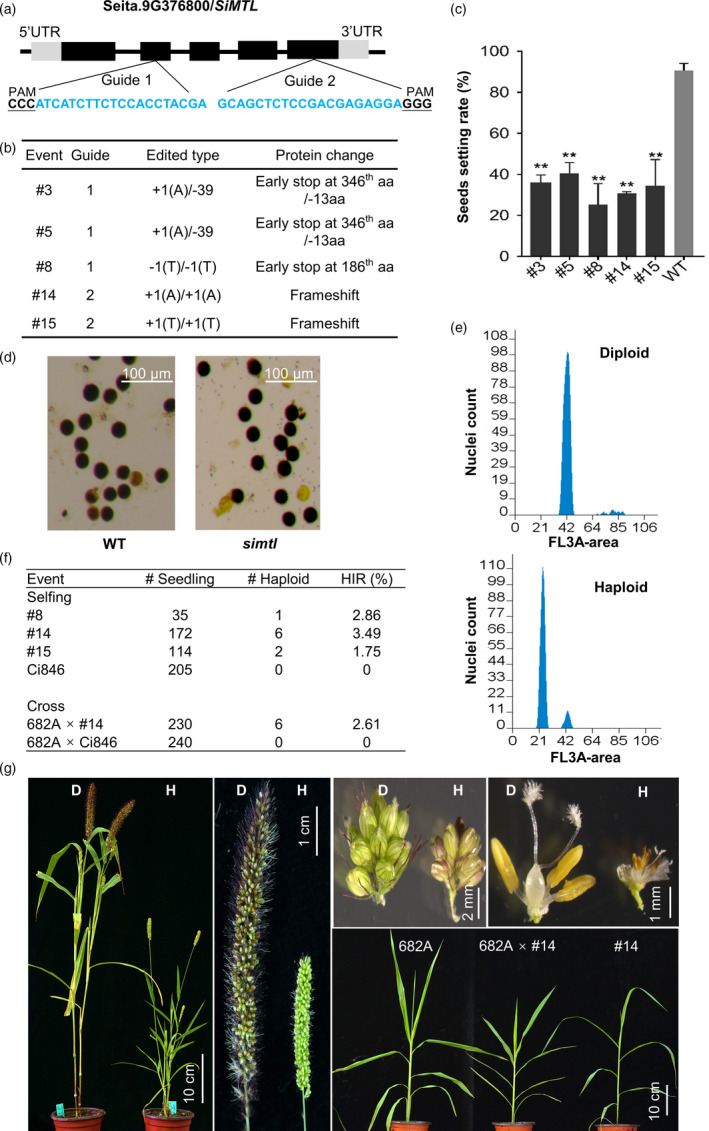
Haploid embryo induction in foxtail millet. (a) Genomic structure of *SiMTL* and targeted sites for the CRISPR‐Cas9 system. PAM is underlined and the guide RNA sequence is highlighted in blue. (b) Five representative T_0_ plants edited at guide 1 or guide 2 in *SiMTL*. The predicted consequence at the protein level resulting from each type of editing is given. (c) Seeds setting rate of the same 5 events. Ten T_1_ plants were grown for each event. For #3 and #5, only homozygous segregants of +1(A)/+1(A) were used for data collection. **Significant difference between *simtl* and WT at *P* < 0.01 by two‐tailed t‐test. (d) Representative pollens from a *simtl* plant, showing normal starch accumulation relative to WT. (e) Verification of haploid plant by flow cytometry analysis. The *x*‐axis indicates the signal peak for nuclei and *y*‐axis indicates the number of nuclei. (f) Haploid induction efficiency (HIR%) determined by self‐pollination or crossing. For crossing, a male sterile line 682A was used as the female and was pollinated with the #14 line. (g) Haploid plant with reduced height and smaller organs relative to WT. A representative haploid plant from crossing, morphologically similar to the female parent, is shown in the lower right panel.

Our result demonstrates that haploid induction can be achieved in foxtail millet simply by knocking out *SiMTL*. Since different inducer lines have variable HIR learnt from maize, we expect that HIR can be improved by creating *simtl* lines with different genetic backgrounds or in combination with mutating other genes using the CRISPR‐Cas9 approach in foxtail millet (Jacquier *et al.,* 2020). Since thousands of hybrid seeds can be easily produced with the improved crossing method (Jiang *et al.,* 2013), it is anticipated that dozens to hundreds of haploid embryos could be obtained at the current HIR for research use. Sorting of tiny seeds with haploid embryos can be facilitated by introducing a fluorescent marker to the inducer, as demonstrated in other plants. In addition, haploid induction‐mediated gene editing has been reported in maize (Kelliher *et al.,* 2019). It is promising that the foxtail millet inducers carrying CRISPR‐Cas9 can be used for such a gene editing strategy. Nevertheless, our successful haploid embryo induction should boost foxtail millet as a C4 model and sheds light on the way to utilize DH in breeding programmes upon further improvement of HIR.

## Conflicts of interest

The authors declare no conflict of interest.

## Author contributions

Y.S., C.W., X.D., Y.G. and X.L. designed the experiments; Y.S., Z.C. and Y.S. performed the experiments; S.Y. conducted flow cytometry analysis; H.Z. made crosses; HS.Z., T.Y. and X.M. provided technical support; Y.S. and C.W. wrote the manuscript.
